# How Does a Sport Psychological Intervention Help Professional Cyclists to Cope With Their Mental Health During the COVID-19 Lockdown?

**DOI:** 10.3389/fpsyg.2021.607152

**Published:** 2021-03-23

**Authors:** Maurizio Bertollo, Fabio Forzini, Sara Biondi, Massimiliano Di Liborio, Maria Grazia Vaccaro, Emmanouil Georgiadis, Cristiana Conti

**Affiliations:** ^1^Behavioral Imaging and Neural Dynamics (BIND) Center, Department of Medicine and Aging Sciences, University “G. d'Annunzio” of Chieti-Pescara, Chieti, Italy; ^2^School of Social Sciences and Humanities, University of Suffolk, Ipswich, United Kingdom; ^3^Independent Psychologist, Rome, Italy; ^4^Department of Medicine and Surgery, University Magna Græcia, Catanzaro, Italy

**Keywords:** sport psychology, mental health, cycling, well-being, performance, professional athletes, elite athletes, coronavirus

## Abstract

All around the world in March, due to COVID-19, competitive sport calendars were suddenly canceled, jeopardizing the training programs of athletes. Moreover, in Italy, the government banned all non-essential travel across the entire country from the beginning of March. Consequently, Italian cyclists were banned from leaving their homes and therefore unable to perform their ordinary training activities. The Italian Association of Professional Cyclists (ACCPI) early on during that period noticed that several cyclists were experiencing a worrying decrease in their mental well-being and asked the authors to set up an online Sport Psychology Intervention (SPI) during lockdown to enhance the athletes' mental health. Through a number of unprecedented events and considerations, the aim of the current investigation was to assess the Italian cyclists' mental health during the lockdown and its changes after the SPI. We validated the Italian version of the Sport Mental Health Continuum Short Form (Sport MHC-SF)—presented in Study 1—and then applied it to a sample of Italian professional cyclists—presented in Study 2—prior to and after the SPI. To achieve these objectives, the reliability and construct validity of the Italian version of the Sport MHC-SF were tested in Study 1. RM-MANOVA tests were run to evaluate the effect of SPI on cyclists in Study 2. A total of 185 Italian athletes were involved in the validation of the MHC in Study 1 and 38 professional cyclists in Study 2. Results from Study 1 suggested a three-factor higher order model of Sport MHC-SF [Model fit: χ^2^(df) = 471.252 (252), *p* < 0.000; CFI = 0.951; RMSEA = 0.049; RMR= 0.048]. MCFA showed that the default model kept invariance among groups of athletes (i.e., female, male, individual, and team sports). Results from Study 2 highlighted that professional cyclists who followed the SPI were able to cope better with psychological stressors, showing improved well-being compared to the athletes that did not. No significant differences were found for emotional and social well-being. The present multi-study paper contributes to the theoretical field with a validated measure of Sport MHC-SF translated in the Italian language and culture. It also provides practical implications related to cases of reduced mental health due to injury, illness, or similar situations of home confinement in the future.

## Introduction

Early in March 2020, Italy became the first country in Europe to enter nationwide lockdown due to the spread of the COVID-19. As a result of emergency legislation by the Italian government, officials closed schools, museums, and theaters and stopped religious and sporting events. All competitive calendars were suddenly canceled, jeopardizing athletes' training preparation. Moreover, the Italian Prime Minister banned all nonessential travels across the entire country from March 10 onwards (DPCM, 2020). Italian athletes had to terminate their training sessions immediately. Cyclists in the same way were banned from leaving their home environment and were also unable to perform their ordinary training activity and volume. Asian athletes had already experienced this outbreak some weeks before, and many other athletes in Europe and other continents followed suit, coping with the effect of the COVID-19 pandemic and having to reorganize their training volume and intensity. Training volume of male professional cyclists (for elite athletes with a professional status) ranges from 16 to 28 h per week for road cyclists and from 14 to 18 h per week for mountain bikers (Sassi et al., 2008). Training volume data of female professional cyclists are still lacking in the literature. However, according to data reported by Sanders et al. (2019) (~18,000 km cycled per year at a mean speed of 28 km h^−1^), weekly volume can be estimated at around 12–16 h for female road professionals. Both men and women cyclists usually compensate on any restrictions to train outdoors due inclement weather (e.g., due to snow, storm, hail) by using Cycling Indoor Training Equipment (CITE) like stationary ergometers, turbo trainers, and rollers. However, CITE is an option normally utilized for no more than a few consecutive days with cyclists resuming their ordinary outdoor training at the earliest opportunity. Of note, differences in terms of biomechanics (Duc et al., 2006; Bertucci et al., 2007), thermoregulation (Brown and Banister, 1985), skin temperature, and heart rate (Mieras et al., 2014) exist between cycling in outdoor conditions and cycling using CITE. Consequently, cyclists cannot perform all their training regimes on CITE since a large volume is required to achieve an elite level in cycling (Jeukendrup et al., 2000). Due to the aforementioned limitations, training sessions on CITE usually last no more than 2 h and are not commonly employed to perform the same workouts that are performed outdoors. Indeed, training specificity is advocated to optimize training adaptation (Millet et al., 2011), and in professional cycling, both high intensity and high volume of training have been shown to be crucial for improving specific peripheral adaptations (Bishop et al., 2014), which have been associated with high-level endurance performance (Jacobs et al., 2011). In this way, to the best of our knowledge, professional cyclists do not adopt CITE as their standard routine of training.

During lockdown, Italian professional cyclists started training on CITE into their houses due to the ban on outdoor training. They were the first cyclists to face the lockdown while their foreign rivals and teammates were still freely training outdoors in their home countries. Moreover, the lockdown affected their ordinary lifestyle, which is characterized by traveling continuously both along short (e.g., going to a cyclist's masseur, to bicycle mechanics, to perform fitness testing, to get a medical examination) and long distances (e.g., participation in their team training camps, travels to test race courses, and eventually going to the competitions). As a consequence, Italian professional cyclists started to experience gradually the adverse effects of lockdown on their mood and mental health. The Italian government started easing the lockdown from May 4 when people were allowed to travel outside of their municipality limits—using also bicycles—but continuing to respect social distancing. Thus, even though collective workouts were still banned, cyclists could start training following individual sessions on the road/off road. Overall, Italian cyclists spent 55 days confined in their homes.

Issues relating to lack of communication with partners and teammates, feelings of isolation, and limited or no access to appropriate contextualized training were taking their toll (Schinke et al., 2020). Specifically, the COVID-19 crisis increased athletes' perceived stress and dysfunctional psychobiosocial states, while decreasing functional psychobiosocial states. Women reported higher perceived stress and dysfunctional states scores than men (di Fronso et al., 2020). Moreover, athletes with higher Athletic Identity tend to ruminate and to catastrophize more, and for this reason, professional athletes may benefit from psychological support provided by sport psychologists in interventions related to cognitive-emotional regulation strategies (Costa et al., 2020). Despite the positive mental health benefits of sport, a number of elite athletes experienced mental health issues during the COVID-19 pandemic mainly due to isolation (Seçkin et al., 2020). This is a new challenge for psychological services and “personal feelings of alienation and poor coping responses, in this case, compounded by social isolation, have resulted for some in compromised mental health” (Schinke et al., 2020, p. 271). For instance, in their scoping review, Kuettel and Larsen highlighted that adverse life events can be mental health risk factors for athletes (Kuettel and Larsen, 2019).

During the COVID-19 pandemic, those risk factors that were related to athletes' personal domain under *normal* circumstances became environmental risk factors potentially able to overthrow protective mechanisms such as feelings of autonomy. Athletes' mental health issues started to become a prominent matter in the sport medicine and sport psychology literature even before the COVID-19 pandemic. Recent pandemic as a “new reality” has created an additional challenge for sporting people. Indeed, sport participation has many benefits, but “the very nature of competition can provoke, augment, or expose psychological issues in athletes” (Chang et al., 2020, p. 91). The mental health of athletes, coaches, and staff is an important component of high performance in sports. According to Poucher and colleagues, the definition of mental health presented by the World Health Organization as “a state of well-being in which every individual realizes his or her own potential, can cope with the normal stresses of life, can work productively and fruitfully, and is able to make a contribution to her or his community” (Poucher et al., 2019). Indeed, mental health not only is the absence of mental illness but also includes the presence of positive feelings (emotional well-being) and positive functioning in individual life (psychological well-being) and community life (social well-being).

High-performance sport environment can help to promote mental health, to destigmatize mental health challenges, to normalize seeking care, and to help with the early identification of a mental health disorder. However, various high-performance sport contexts consist of a unique range of stressors such as competitive factors (e.g., performance expectation) and organizational (e.g., travel) and personal stressors (e.g., family issues) that potentially increase athletes' risk for mental illness (Sarkar and Fletcher, 2014; Rice et al., 2016). Unique stressors associated with sport participation in elite athletes include overtraining, pressure to perform, poor athletic performances, coach–athlete or teammate–athlete relational tensions, injury, and stressors related to retirement (Noblet et al., 2003; Bruner et al., 2008).

Many elite athletes experience depression, anxiety, and other psychological disorders, and they do not seek specialized and efficient treatment (e.g., Schinke and Stambulova, 2017; Moesch et al., 2018). Lack of time, limited knowledge around mental health, and negative help-seeking experiences in the past (Gulliver et al., 2012) are important barriers for seeking help for a mental disorder. The primary barrier perceived by the athletes is the stigma associated to mental health issues (Bird et al., 2018).

During most of the twentieth century, the concept of mental health has been largely undefined and related to the existence or absence of mental illness (Smith, 1959). However, following the appreciation that significant “dimensions of psychological well-being are intra-reflections of an individual's adjustment to and outlook on their life,” Keyes (2002, p. 209) suggested that caring for mental health promotion rather than treating mental illness is the key priority on mental health. He proposed 13 dimensions of flourishing mental health that have been seminal in defining and measuring mental health even today (Keyes, 2007).

Keyes' suggestions on what he referred to as the “Mental Health Continuum,” currently denoted as the Mental Health Continuum—Long Form (MHC-LF), comprises a comprehensive measurement instrument encompassing all three dimensions of well-being (subjective, psychological, and social) (Keyes, 2002). The measure consists of 40 items (7 items measuring subjective well-being and positive affect, six 3-item subscales measuring the six dimensions of psychological well-being, and five 3-item subscales measuring the five dimensions of social well-being). Well-being and mental health are relevant factors to maintain peak performance in athletes as mental health is not only the absence of illness or dysfunction but also the presence of subjective well-being. Based on this concept, “mental health is a continuum, not a discreet fixed state of mind or mood where an athlete spends his entire life (Lardon and Fitzgerald, 2013, p. 133).”

The above—original—Keyes' scale has been contracted into a short form (MHC-SF) with its psychometric properties later evaluated by Lamers et al. (2011). With the purpose of adopting the MHC-SF in the sporting context, Foster and Chow (2018) created a sport-specific form of the MHC-SF (Sport MHC-SF), which is composed of 14 items and three subscales (i.e., psychological well-being, social well-being, and subjective well-being). The Sport MHC-SF is a sport-specific instrument, which has been demonstrated to be a valid and reliable measure of sport well-being through Confirmatory Factor Analysis (CFA), internal consistency, and discriminant validity analyses (Foster and Chow, 2018). However, an Italian version of the Sport MHC-SF is not yet available.

Early on during the COVID-19 lockdown, the Managing Council of the Italian Association of Professional Cyclists (ACCPI) reported that several cyclists were experiencing a worrying decrease in their mental well-being and asked to set up an online Sport Psychological Intervention (SPI) during the lockdown to enhance the athletes' mental health. The SPI was supervised and led by the first author with the collaboration of four psychologists with strong expertise in the sport field. All collaborators are psychotherapists and addressed to the Italian professional cyclists after the first 3 weeks of lockdown.

Based on the abovementioned urgencies and necessities, the aim of the current study was to assess the Italian cyclists' mental health (in terms of subjective well-being) during the lockdown and its changes after the SPI. In Study 1, we created and validated the Italian version of the Sport MHC-SF, and then in Study 2, we applied it to a sample of Italian professional cyclists prior to and after the SPI.

## Study 1

The purpose of Study 1 was to culturally adapt and validate the Sport MHC-SF (Foster and Chow, 2018) in the Italian language and context. Before lockdown, between September and December 2019, the authors collected the data to validate the instrument that have potential in clinical practice. Following that period, it has been beneficial in the assessment phase of the professional cyclists involved in our study during lockdown.

### Methods

#### Participants

A total of 185 Italian athletes (89 men, 96 women) who ranged in age from 18 to 52 years (*M* = 26.95; SD = 8.40) agreed to participate in this study. The sample size is sufficient to ensure a person-to-item ratio of 10:1 (Cabrera-Nguyen, 2010). Participants were involved in individual (*N* = 93) and team sports (*N* = 92), such as climbing (*n* = 1), kite surfing (*n* = 1), combat sports (*n* = 3), fencing (3), gymnastics (*n* = 3), triathlon (*n* = 5), boxing (*n* = 4), water polo (*n* = 5), softball (*n* = 6), powerlifting (*n* = 6), skiing (*n* = 8), tennis (*n* = 9), swimming (*n* = 9), basketball (*n* = 11), futsal (*n* = 13), martial arts (*n* = 16), soccer (*n* = 21), cycling (*n* = 25), and volleyball (*n* = 36). They reported participating in 3 to 10 training session per week. Four levels of sport participation were reported, namely, local (*n* = 20), regional (*n* = 60), national (*n* = 65), and international (*n* = 40) level.

#### Materials and Instruments

The *Sport Mental Health Continuum—Short Form* (Sport MHC-SF; Foster and Chow, 2018). The Sport MHC-SF represents the adaptation of the Mental Health Continuum—Short Form (MHC-SF; Keyes et al., 2008) in the sport domain. The Sport MHC-SF assesses the constructs of emotional, social, and psychological well-being in sport. The scale comprises 14 items divided into three factors, namely, sport emotional well-being (Items 1–3), sport social well-being (Items 4–8), and sport psychological well-being (Items 9–14). Each item is responded on a six-point Likert scale, ranging from 0 (=never) to 5 (=every day). CFA showed a clear three-factor structure and internal consistency reliability was 0.89 for the subjective well-being subscale, 0.88 for the social well-being subscale and 0.90 for the psychological well-being.

The *Italian Mood Scale* (*ITAMS*; Quartiroli et al., 2017) measures the mood responses, and it is the validated Italian version of the Brunel Mood Scale (BRUMS; Terry and Lane, 2010). The 24-item ITAMS is divided into six factors, namely, Anger, Confusion, Depression, Fatigue, Tension, and Vigor. The response of each item was rated on a five-point Likert scale ranging from 0 (=not at all/per nulla) to 4 (=extremely/moltissimo).

The *12-item Short Form Health Survey*, Italian version (*SF-12*, Apolone et al., 2001). The original SF-12 is a generic measure of health status, and it was developed by Ware et al. (1996) as the shorter version of the *36-item Short Form Health Survey* (*SF-36*, Ware et al., 1994). The 12 items are included into two subscales, namely, Physical Component Summary (PCS) and Mental Component Summary (MCS). We have used the validation of the Survey in Italian language (Apolone et al., 2001).

#### Procedures

Before lockdown, between September and December 2019, a group of bilingual (i.e., two Italian and two English) experts in sport and clinical psychology translated the Sport MHC-SF following a standard procedure based on the forward–backward translation method (Beaton et al., 2000). After discussing a few minor discrepancies on the syntax to establish agreement, we drafted the Italian version of the Sport MHC-SF in sport (see Appendix 1).

Participants were recruited using the authors' informal and professional network (i.e., clubs and sport centers). Coaches at these locations were informed regarding the study details (purpose and methodology) and identified any athletes who may have met eligibility criteria of potential study participation (i.e., more than 18 years old and currently active in competitions). The study details were explained to the eligible athletes, and they gave informed consent agreeing to participate. Personal data were hidden, and all participants were anonymized. The study was conducted in accordance with the Declaration of Helsinki, and participants signed the informed consent to participate in the study. Each participating athlete completed the Sport MHC-SF, the ITAMS, and the SF-12 in about 20 min.

#### Statistical Analyses

Data were initially screened for multivariate outliers and normal distribution (Tabachnick and Fidell, 2013). Examination of histograms, skewness, and kurtosis of the variable scores showed that there were no substantial deviations from normal distributions.

We investigated the construct validity, the internal consistency, and concurrent validity of the Sport MHC-SF. To explore the factor structure of the scale and the invariance across the four groups of our sample (i.e., female, male, individual, and team sport), a Multigroup Confirmatory Factor Analysis (MCFA) was performed using AMOS graphic (Version 24.0; IBM, Armonk, NY, USA) in order to establish the comparability of measurement models across independent samples (Milfont and Fischer, 2010). We estimated CFA models using the maximum likelihood parameter estimates with standard errors and a mean-adjusted chi-square test statistic that is robust to non-normality. The models were evaluated according to different fit indices: root mean square residual (RMR), root mean square error of approximation (RMSEA), Tucker–Lewis fit index (TLI), and comparative fit index (CFI). Generally, an acceptable fit is inferred when RMR and RMSEA values are <0.08 (Hu and Bentler, 1999; Hooper et al., 2008). TLI and CFI values <0.90 are also considered reflective of good fitting models (Tucker and Lewis, 1973). The CFA model was evaluated according to the following fit indices: the chi-square test (χ^2^), the CFI, the TLI, the RMSEA, and the standardized root mean square residual (SRMR). We followed the guidelines proposed by Hu and Bentler (1999), (i.e., the values of CFI close to 0.95 or above, RMSEA close to 0.06 or below, and SRMR close to 0.08 or below as indications of good fit, using these indices in combination; Brown, 2015).

Modification indices were also inspected to improve the model, and meaningful associations were included. Moreover, A step-up approach was adopted to examine different forms of invariance (i.e., configural, metric, scalar) following the suggestion by Crowson (2020). A baseline model was computed by gathering scores from female and male athletes participating in individual and team sports, and the expected relationships between each item and their latent factor were checked (i.e., configural invariance). Metric and scalar invariances were then, respectively tested by constraining the factor loadings and intercepts of items to be the same across samples. The comparisons of root mean square error of approximation (ΔRMSEA) and comparative fit indexes (ΔCFI) were used to inspect the changes in goodness of fits across models. A ΔCFI ≤ 0.01 and a ΔRMSEA ≤ 0.015 indicate that the null hypothesis of invariance should not be rejected (Chen, 2007).

The reliability of each Sport MHC-SF subscale was assessed in terms of internal consistency (Cronbach's α). However, as alpha has been criticized by methodologists as an inappropriate measure of internal consistency reliability (Dunn et al., 2014), we also present McDonald's omega, calculated using MBESS package in R. Additionally, concurrent validity was determined using a Pearson product moment correlation (Pearson *r*) between subscales of Sport MHC-SF, subscales of ITAMS, and subscales of SF-12.

### Results

**Figure 1:**
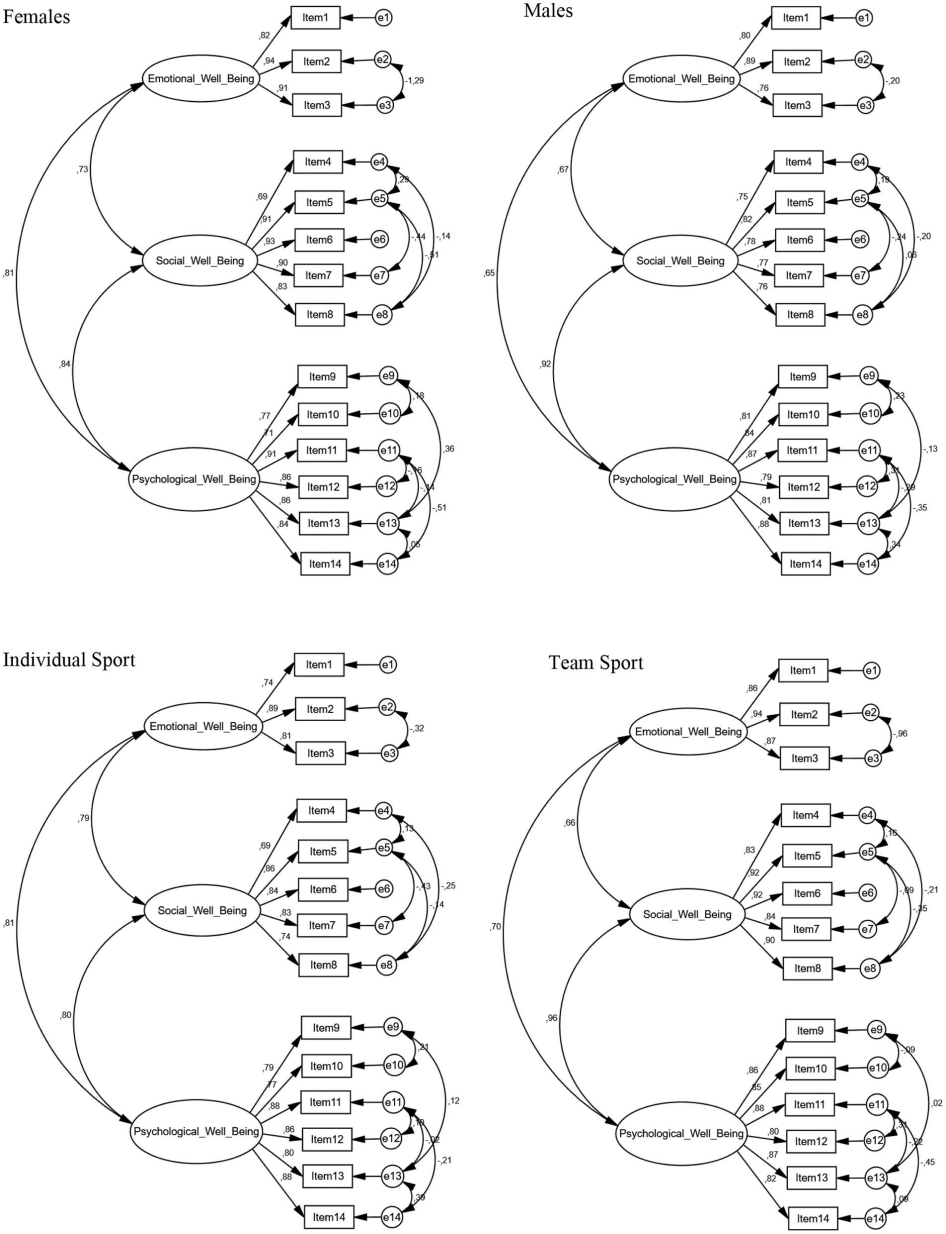
Multigroup Confirmatory Factor Analysis of the Italian version of the Sport MHC-SF scale: standardized regression weight estimates.

The scale demonstrated good reliability in terms of internal consistency (Cronbach alpha) for the aggregate score (α = 0.90) and good to excellent reliability for the independent scores (“emotional well-being” = 0.85; “social well-being” = 0.90; “psychological well-being” = 0.93). We obtained similar results also for McDonald's omega (emotional well-being Ω = 0.86, 95% CI = 0.82, 0.90; social well-being Ω = 0.90, 95% CI = 0.87, 0.93; psychological well-being Ω = 0.94, 95% CI = 91, 0.96). Multigroup Confirmatory Factor Analyses (MCFAs) evidenced a very good fit for the model proposed by Foster and Chow (2018) with “sport emotional well-being,” “sport social well-being,” and “sport psychological well-being” as dimensions [Model fit: χ^2^(df) = 471.252 (252), *p* < 0.000; CFI = 0.951; RMSEA = 0.049; RMR = 0.048]. Factor loadings for each item were significant and standardized regression weights ranged between 0.74 and 0.94 for the emotional well-being subscale, between 0.69 and 0.93 for social well-being, and between 0.71 and 0.91 for psychological well-being (see Figure 1 for full details). Regarding measurement invariance among the male, female, individual, and team sport samples, when constraining factor loadings and intercept of items across groups, we obtained ΔCFI = 0.001, RMSEA = 0.003 for metric invariance and ΔCFI = 0.004, ΔRMSEA = 0.001 for scalar invariance. These findings suggested that measurement invariance could be established among the samples (Cheung and Rensvold, 2002; Chen, 2007).

Correlation among Sport MHC-SF subscales, ITAMS subscales, and SF-12 subscales showed significant positive values between the subscales of Sport MHC-SF and SF12 subscales as well as with the vigor subscale of ITAMS. On the other hand, negative correlations emerged between Sport MHC-SF scales and the negative subscales of ITAMS (i.e., anger, confusion, depression, fatigue, tension). However, the values of correlation were low, confirming that Sport MHC-SF provide complementary information to mental health in athletes (see Table 1).

**Table 1:** Concurrent validity among Sport MHC-SF subscales, ITAMS subscales, and SF-12 subscales.

**Latent variable**	**(1)**	**(2)**	**(3)**	**(4)**	**(5)**	**(6)**	**(7)**	**(8)**	**(9)**	**(10)**	**(11)**
(1) Emotional well-being											
(2) Social well-being	0.620										
(3) Psychological well-being	0.651	0.810									
(4) Anger	−0.213	−0.103	−0.085								
(5) Confusion	−0.189	−0.143	−0.170	0.597							
(6) Depression	−0.249	−0.164	−0.203	0.694	0.718						
(7) Fatigue	−0.143	−0.060	−0.081	0.490	0.505	0.466					
(8) Tension	−0.190	−10.63	−0.172	0.605	0.633	0.642	0.526				
(9) Vigor	0.352	0.337	0.351	−0.083	−0.199	−0.226	−0.232	−0.152			
(10) PCS	0.202	0.196	0.252	−0.171	−0.208	−0.252	−0.114	−0.104	0.237		
(11) MCS	0.359	0.350	0.358	−0.348	−0.434	−0.509	−0.377	−0.572	0.392	0.092	

*All correlation coefficients are significant at p < 0.01*.

To provide a more detailed overview of the level of mental well-being of the sample of the Italian athletes, we compared the data of our sport-specific sample with similar measures (i.e., traditional version of MHC-SF) collected with an Italian normal population and reported in Petrillo et al. (2015). Effect size of the mean differences was estimated using Cohen's *d* (Cohen, 1988), for which 0.20, 0.50, and 0.80 are considered small, medium, and large effects, respectively. Specifically, the means (and standard deviations) of emotional, social, and psychological well-being observed during lockdown were compared with those provided in Study 1 (see Table 2). We performed this comparison as the literature provides contradictory results. Moesch et al. (2018) reported that similar rates of mental issues exist when comparing athletes to normal populations. Lundqvist and Sandin (2014) have suggested the need to define more explicitly the conceptual framework of well-being in elite sport. Furthermore, Henriksen et al. (2020) recommended to better define mental health in a sport context and broaden the scope of assessment in sport-related mental health research.

**Table 2:** Mean and standard deviation of the Italian and normal population in mental health dimensions.

**Mental health dimension**	**Italian normal population**		**Italian sport population**		**Cohen *d***
	**M**	**SD**	**M**	**SD**	
Emotional well-being	3.97	1.12	3.85	0.96	0.07
Social well-being	2.73	1.01	3.90	1.13	1.09
Psychological well-being	4.45	1.02	3.97	0.97	0.48
Total well-being	3.73	0.85	3.91	0.91	0.21

### Discussion

Study 1 aimed to provide an adaptation and validation of the *Sport Mental Health Continuum—Short Form* (Sport MHC-SF; Foster and Chow, 2018) in Italian language. Therefore, we investigated the construct validity, the internal consistency, and the concurrent validity of the scale in a sample of competitive Italian athletes.

The Italian version of Sport MHC-SF displayed a clear factorial structure and reflects the original version of the scale. Indeed, findings displayed the theoretically based arrangement of the 14 items in the three subscales: sport emotional, sport social, and sport psychological well-being. The CFA indicated an excellent fit for the model of the multidimensional structure of athletes' well-being, and that the loadings of all dimensions on the higher-order factor for mental health were reasonable.

The Italian version of Sport MHC-SF showed a very good internal consistency, similarly to the original scale and to the MHC-SF (Lamers et al., 2011). Cronbach alpha values exceeded 0.85 for all three subscales. Well-acceptable results on reliability were also obtained through McDonald's omega scores.

Concurrent validity analysis indicated that the Italian version of Sport MHC-SF is correlated with ITAMS subscales and SF-12 subscales. The positive correlation with the SF-12, and particularly with the Mental Component Summary, is encouraging considering that it is a generic measure of health status. Moreover, findings showed that the three questionnaires share some common variance, but at the same time, they measure different constructs.

It is also important to highlight the differences between the normal and sport population. Also, if the instruments used are slightly different in some items (see Appendix 1), conceptually they measure the same constructs (i.e., emotional, social, and psychological well-being). Although we found very small differences in total well-being and no differences in emotional well-being, moderate and large differences between the samples emerged for social and psychological well-being, with higher level of social well-being and lower level of psychological well-being in athletes. This is partially in line with the contradictory literature that reports similar rates of mental issues in athletes compared to the normal population (Moesch et al., 2018) or that “elite athletes experience a broadly comparable risk of high-prevalence mental disorders (i.e., anxiety, depression) relative to the general population” (Rice et al., 2016, p. 1333) or that psychological well-being is better in athletes compared to non-athletes (Alamdarloo et al., 2019). However, Lundqvist and Sandin (2014) suggested the need to define more explicitly the conceptual framework of well-being and the level (global or context-specific) on which the construct is investigated. Their integrated model of global well-being and context-specific well-being related to sport can be examined further in the future.

As mentioned before, a reliable tool for the evaluation of mental health levels in a sporting environment was not available in the Italian language and context. Hence, the development of the Italian version of the Sport MHC-SF allows professionals to examine Italian athletic populations' well-being and share projects and results with scholars in other countries. In conclusion, the Italian version of Sport MHC-SF is suggested to be a valid scale in the evaluation of the athletes' well-being and specifically for measuring the variability of well-being levels among athletes, with reference to emotional, social, and psychological variables. However, some limitation should be highlighted such as its small sample size and the need of a larger validation study checking for competitive level invariance.

## Study 2

The purpose of Study 2 was to analyze the effects of SPI based on a mental health literacy program of professional cyclists during the COVID-19 lockdown, describing a professional intervention commissioned by the ACCPI.

### Materials and Methods

#### Participants

A total of 38 Italian professional cyclists (10 men, 28 women) who ranged in age from 18 to 37 years (*M* = 25.11; SD = 4.87) agreed to participate in this study. They reported participating in 6–12 training sessions per week. Athletes were all competing at international level with an average of 4 h of training per day.

During the design of the SPI, all cyclists supported by the ACCPI were invited to participate. Nineteen athletes agreed immediately to take part in the SPI to enhance their mental health during the lockdown. Following that, another group of 19 cyclists asked to be included in the program and they formed a waiting list for the current study. Comparison between those two groups made sense since the comparison group spent equal time dealing with generic sport psychology skills, while not focusing on mental health literacy. It was our best chance to compare a carefully crafted intervention aiming mental health components to generic (or performance based) interventions within the elite sport domain. Therefore, we had two groups of professional cyclists: 19 athletes involved in the SPI group and 19 athletes (forming the waiting list) who were included in the comparison group.

#### Instruments and Intervention

The Sport Mental Health Continuum-Short Form (Sport MHC-SF; Foster and Chow, 2018), described in Study 1, was administered to analyze emotional, social, and psychological well-being in the participating cyclists. Between the two Sport MHC-SF assessments, a specific SPI was conducted with the athletes that agreed to participate.

The SPI was requested during the COVID-19 lockdown from the President of the ACCPI to prevent decreases in the athletes' mental well-being. The SPI was proposed and led by the first author with the collaboration of four psychologists and psychotherapists with strong expertise in the sport field. Intervention with the cyclists initiated about 3 weeks after the initial point of lockdown. Authors designed the intervention responding to components included in recent definitions of Mental Health Literacy (Furnham and Swami, 2018) with an emphasis on sport-specific needs and priorities (Gorczynski et al., 2020).

Indeed, mental health literacy and educational strategies are fundamental in improving mental well-being. Moreover, we took into consideration the ecological framework of elite athletes' mental health needs proposed by Purcell et al. (2019). They suggested that “core foundational components” should include (i) mental health literacy to improve understanding, reduce stigma, and promote early help-seeking; (ii) a focus on athlete development (both career and personal development goals) and skill acquisition to help attain these goals; and (iii) mental health screening of, and feedback to, athletes” (p. 3). Furthermore, we used suggestions from international scholars and organizations (Reardon et al., 2019; Association for Applied Sport Psychology, 2020; International Olympic Committee, 2020a,b).

The SPI consisted of a webinar on mental health and well-being in athletes followed by three online focus groups. A Decalogue of behavioral indications to cope with the lockdown were constructed and introduced during the webinar. The mental health model is the Keyes (2002) two-continuum model, which includes, in one continuum, the presence and absence of mental illness or mental health concerns and, in the other continuum, the presence and absence of mental well-being. Mental health promotion serves as a protective feature against stressors (World Health Organization, 2002) and the International Society of Sport Psychology (ISSP) made a call for sport psychology consultants to increase the promotion of athletes' mental health (Schinke et al., 2018). The comprehensive framework for mental health to support elite athletes (Purcell et al., 2019) aims to reduce the risk of mental health symptoms developing or minimizing their potential impact and severity, informing in the same way our SPI. Such programs broadening mental health literacy help to build a culture that values the importance of mental health and well-being of athletes.

**Figure 2:**
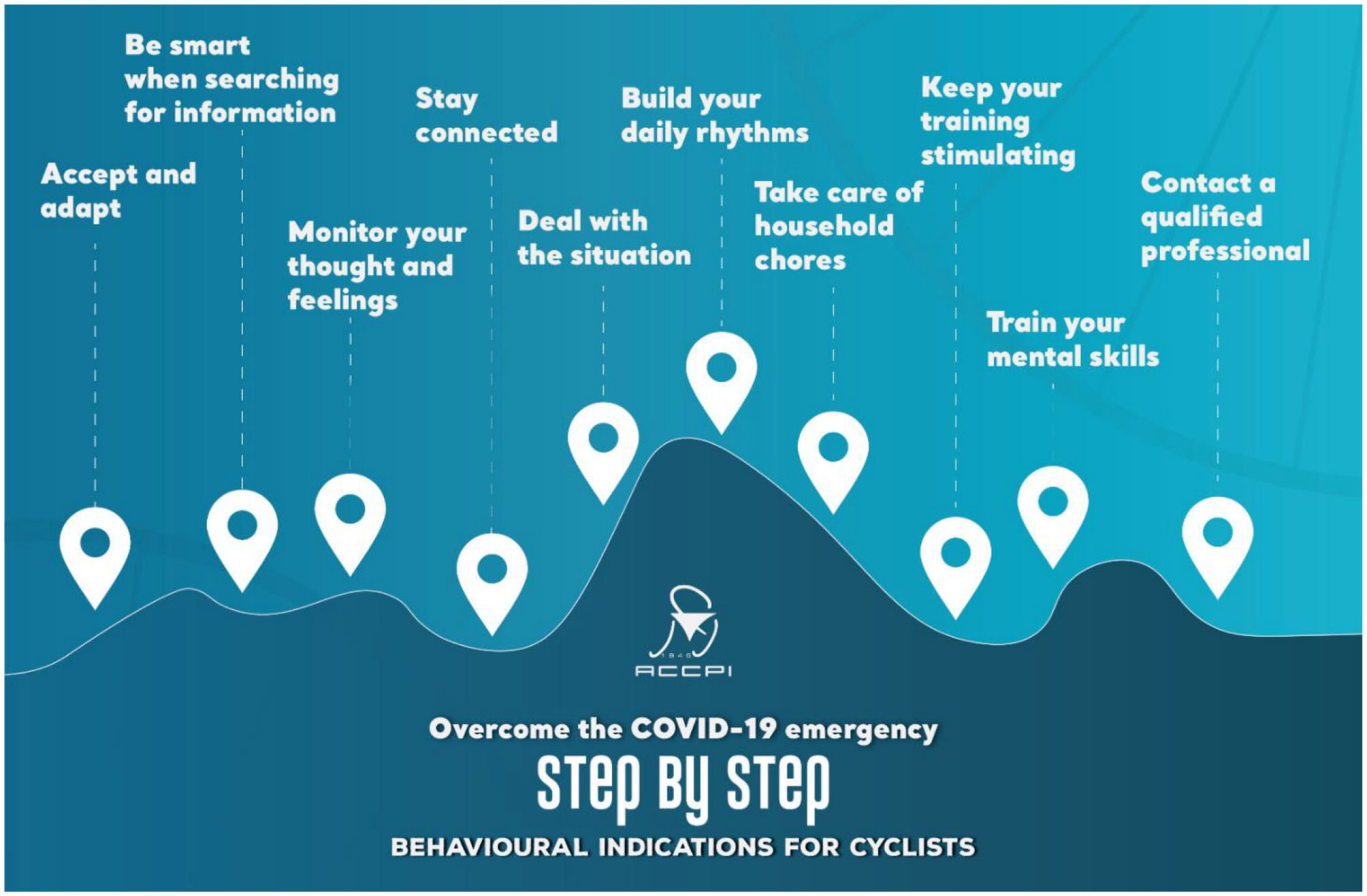
Infographic: Decalogue of behavioral indications for cyclists.

**Figure 3:**
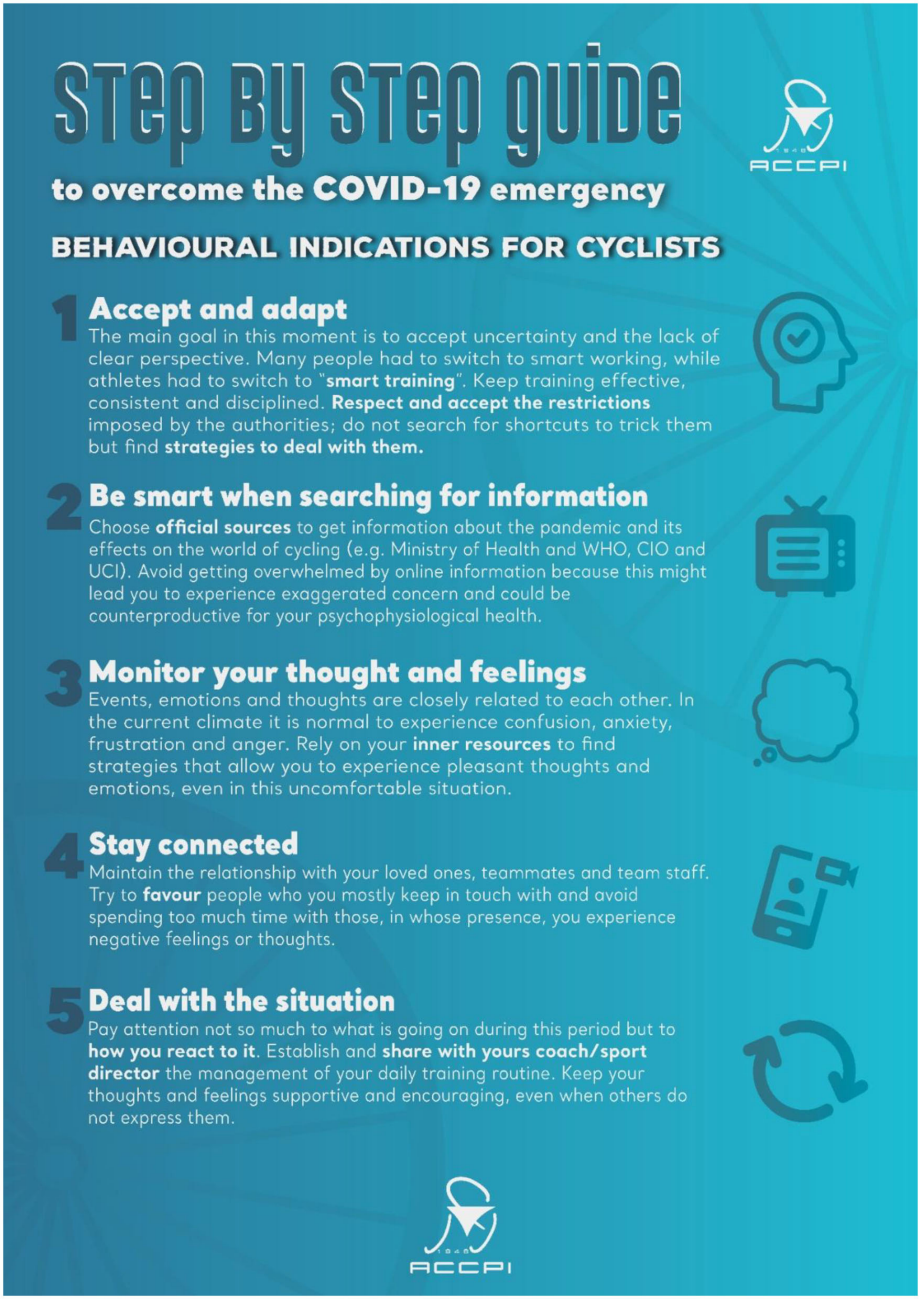

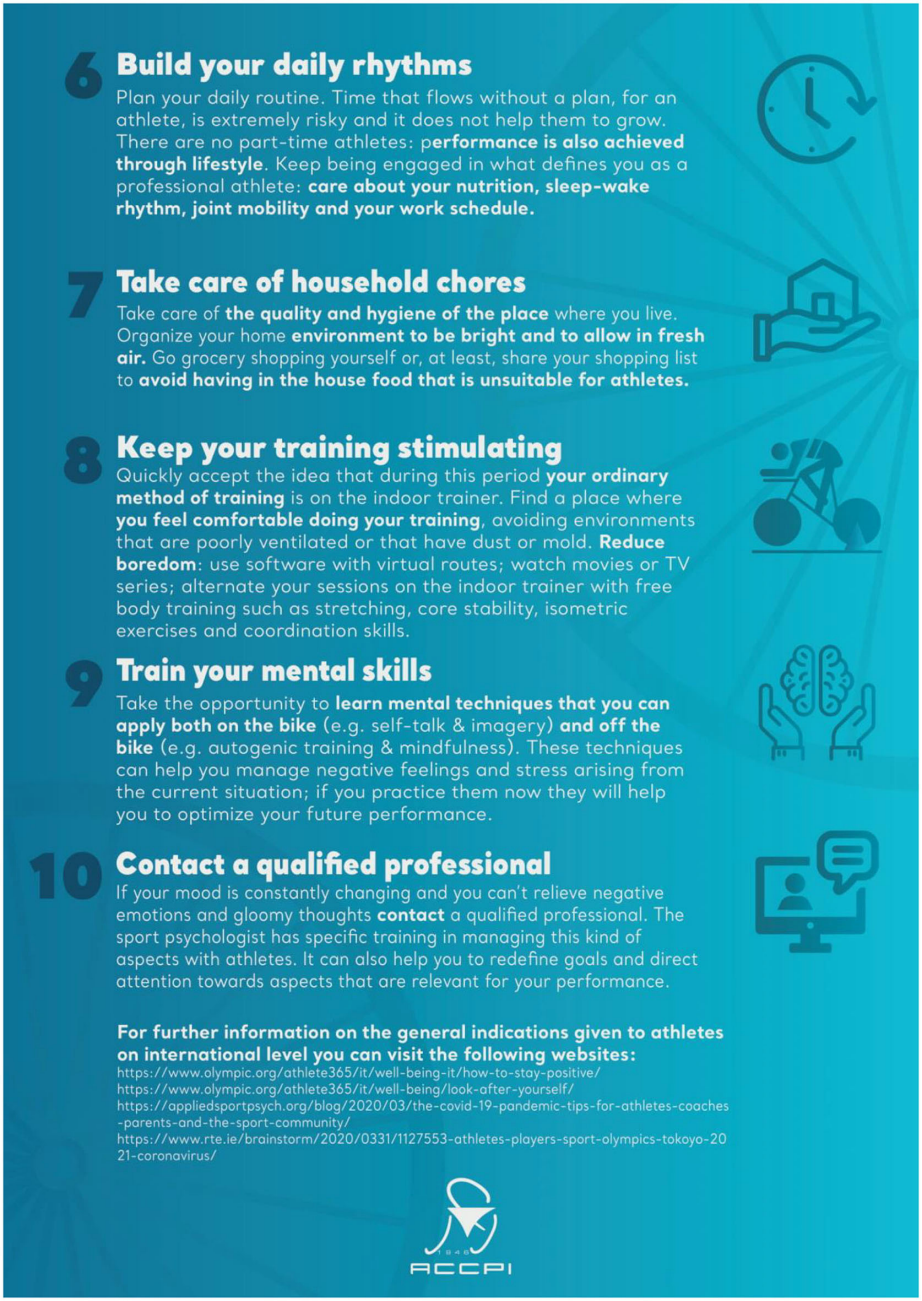
Decalogue of behavioral indications for cyclists.

Authors utilized infographics as an efficient method to construct the Decalogue of behavioral indications during the lockdown for professional cyclists (see Figures 2, 3). Infographics is “an increasingly popular visual approach to communicate information (Muir and Munroe-Chandler, 2020, p. 2),” through the combination of images and text, since they clearly and concisely encapsulate large volumes of information (Dunlap and Lowenthal, 2016). It is also an efficient method to convey complex information to wider audiences promoting concepts and ideal practices (McCrorie et al., 2016).

Infographics can potentially be an important gateway to discuss mental health topics with athletes. Sport psychology consultants can increase athletes' awareness on the importance of mental health and also act as a liaison between athletes and mental health practitioners (Muir and Munroe-Chandler, 2020). In the particular case, the Infographic Decalogue was used as an introductory point for discussing important behavioral recommendations during lockdown. It assisted authors to initiate the discussion on the importance of mental health in professional sports, especially during a critical period such as the COVID-19 pandemic.

Online focus groups were directed on the development of “basic techniques for athletes to self-manage transient mood states or psychological distress, such as relaxation techniques, [and] adaptive coping strategies” (Purcell et al., 2019, p. 5) helping professional cyclists deal with various difficulties (isolation, interruption of sport events, the reduction of the training volume, etc.) during lockdown. More precisely, athletes were presented with self-talk, relaxation, mindfulness, self-efficacy, and imagery skills.

#### Procedure

After the first author was contacted by the ACCPI, an initial screening of the professional cyclists—voluntarily deciding to participate—was performed. ACCPI asked their associated professional cyclists to voluntary participate in the intervention. Thirty-eight cyclists joined the program with the first 19 included in the SPI, whereas the19 athletes of the waiting list constituted the comparison group.

SPI lasted 1 month and was based on mental health literacy intervention. It consisted of:

- Creating and sending to the cyclists an Infographic Decalogue of behavioral guidelines helping them to cope with the lockdown (see Figures 2, 3),
- Conducting a webinar on mental health literacy and behavioral indication, as well as
- Three online focus groups on specific topics mainly devoted to the eudaimonic well-being for positive mental health.

Online workshops were held on a weekly basis, lasting 2 h and using a popular communication platform. During the first meeting, the president and the general secretary of ACCPI introduced the reported project with the five experts underlying the importance of supporting athletes' mental health during this critical period in accordance to the requests of the professional cyclists to their association.

Online focus groups comprised a theoretical introduction to the topic (first part) and a discussion (second part) of the following topics:

1. A mental health webinar was focused on the Keyes (2002) model of mental health continuum and on the introduction and explanation of the Decalogue of behavioral suggestions. Theory followed by the ecological framework by Purcell et al. (2019) was also mentioned to increase cyclists' awareness of mental well-being and decrease the stigma related to mental health issues.
2. The first online focus group entitled “Acting on one's thoughts to change emotions and behaviors: experiences of self-talk” explored self-talk technique from a motivational and attentional point of view. Theory was followed by a discussion with the athletes on the emotional and motivational problems they were facing during the lockdown and the self-talk technique as a strategy to cope with them. Moreover, the basics of Rational Emotive Behavioral Therapy (REBT) in sport have been introduced following the suggestion by Turner et al. (2020).
3. The second online focus group entitled “From body to mind and vice versa: mindfulness and relaxation experiences” offered an overview of relaxation and mindfulness techniques. After a theoretical introduction, a practical session on relaxation (Autogenic Training; Shulz and Luthe, 1969) and mindfulness (Kabat-Zinn, 1982, 1990; Birrer et al., 2012) was presented to the participating athletes.
4. The third online focus group entitled “Self-esteem and Self-efficacy: Imagery experiences” examined the use of imagery as a technique to increase self-esteem and self-efficacy. After a theoretical introduction, a practical session of imagery was presented to the athletes (Holmes and Collins, 2001; Razon et al., 2014).

All the above were directed to the cyclists comprising the intervention group. A month after the end of the intervention, cyclists from both groups filled out the Sport Mental Health Continuum—Short Form (Sport MHC-SF; Foster and Chow, 2018) again. Scores were compared and analyzed accordingly (see below).

#### Statistical Analysis

Data were initially screened for multivariate outliers and normal distribution (Tabachnick and Fidell, 2013). No missing values were identified due to the online submission in which all items had to be rated. To test the between- and within-group effects of the SPI, data were analyzed running a repeated measure multivariate analysis of variance (RM-MANOVA) 2 (group: SPI and comparison) × 2 (time: test and retest) on all the scales of MHC. Effect sizes were calculated using partial eta square (η_p_^2^; Lakens, 2013), with 0.01, 0.06, and 0.14 considered small, medium, and large effects, respectively (Cohen, 1988). The significance level was set at 0.05, and statistical analyses were performed using the SPSS software (Version 25.0; IBM, Armonk, NY, USA).

### Results

Descriptive statistics for the two groups of cyclists are provided in Table 3.

**Table 3:** Descriptive statistics on Sport MHC-SF for the two groups of cyclists.

	**Test**		**Re-test**	
	**SPI group**	**Comparison group**	**SPI group**	**Comparison group**
Emotional well-being	3.92 (0.82)	4.03 (0.96)	3.85 (0.81)	3.64 (0.67)
Social well-being	3.69 (0.86)	4.00 (0.95)	3.57 (1.07)	3.96 (1.35)
Psychological well-being	3.89 (0.67)	4.27 (0.85)	4.14 (0.73)	3.60 (0.87)

According to the suggestion provided by Keyes et al. (2008) and used also for the Italian validation of the MHC-SF in the normal population, we also applied diagnostic categories in the categorical assessment of mental health distinguishing levels of psychosocial functioning between flourishing, moderately mentally healthy, and languishing individuals. We did not find any languishing cyclists in any of the groups but found six moderately healthy athletes in each group. At the end of the lockdown, we found three cyclists who were still moderately healthy individuals in the SPI group with all the other athletes flourishing. Meanwhile, in the comparison group, the same six cyclists remained moderately healthy.

To provide a more detailed overview of the extent to which the COVID-19 lockdown impacted athletes' mental health and well-being, we compared the data of our cyclists before the SPI (see test of SPI group in Table 3) with those of the same measure collected with Italian athletes before lockdown and reported in Study 1 (see Italian sport population in Table 2). Effect size of the mean differences was estimated using Cohen's *d* (Cohen, 1988), for which 0.20, 0.50, and 0.80 are considered small, medium, and large effects, respectively. Specifically, the means (and standard deviations) of emotional, social, and psychological well-being, observed during lockdown, were compared with those provided in Study 1. We found small to no effect on emotional (Cohen *d* = 0.08), social (Cohen *d* = 0.20), and psychological well-being (Cohen *d* = 0.09) for the Intervention group, and similar effect on emotional (Cohen *d* = 0.18), social (Cohen *d* = 0.10), and psychological well-being (Cohen *d* = 0.32) for the comparison group.

We assessed the effect of SPI between and within participants of study 2. RM-MANOVA yielded significant differences only for the group × time interaction on MHC (Wilks λ 0.772, *p* = 0.03, $\eta_{\text{p}}^{2}$ = 0.30, power = 0.71). Univariate follow-up showed significant differences on psychological well-being, *F*_(1,36)_ = 6.91, *p* = 0.012, $\eta_{\text{p}}^{2}$ = 0.17, power = 0.77. Professional athletes who received the intervention reported higher psychological well-being in the retest compared to the athletes who did not follow the SPI. No significant differences were found for emotional and social well-being.

### Discussion

The purpose of Study 2 was to examine the effects of a short educational intervention, i.e., SPI, on professional cyclists during the COVID-19 lockdown. Its aim was to improve psychological responses through a mental health literacy intervention, supporting cyclists' ability to cope with the effects of the confinement. The intervention consisted of an Infographic Decalogue of behavioral guidelines specifically created and sent to the cyclists, along with a webinar on mental health and well-being. Both these interventions were followed by three online focus groups. Due to the practical constraints of lockdown, the online focus group methodology was necessary to allow group discussions among athletes in different geographical regions.

Online interactions may hinder engaged conversations. Hence, psychologists and psychotherapists conducting the intervention decided to maintain the same structure for each focus group: each meeting begun with an introduction of the topic, allowing athletes to feel comfortable while starting to engage in the conversation. In the second part of the online focus groups, the experts encouraged athletes to share views and experiences engaging in conversations or using the group chat. Both modes of interaction supported the elicitation of viewpoints making those focus groups engaging and productive.

The intervention had an effect particularly on the eudaimonic feature of mental health showing important effects on psychological well-being. This is an important finding since it came as a product of a brief intervention limited on certain skills we prioritized based on related literature. Similarly, selected topics supported self-management, personal choice, and autonomy skills during a period of scarce control over one's environment. These topics obviously paid dividends showing participating cyclists reducing their distress and benefiting from those skills, at least during the confinement period.

However, we did not observe any changes in the hedonic (emotional) well-being and the social well-being. Given that the mental health is also an indication of alignment between the individual athlete and the surrounding context (Henriksen et al., 2020), the difficulty of the applied intervention to reach these elements of well-being is self-explanatory. Included skills and techniques did not incorporate areas of importance for this mental health component such as self-compassion and enhanced emotion experiences due mainly to the short duration and the urgency of the intervention (Galla, 2016). Longer and richer interventions could shed more light on such a possibility.

Social well-being relates to the degree individuals see themselves thriving within their social environment (Keyes, 2002). Even if social connection was included in the 10-point infographic, we did not put an emphasis on social connectedness. Again, both the ongoing pandemic and the short duration of the intervention did not allow chances for influencing this mental health component.

Interestingly, cyclists of the comparison group presented a slight decrease on their mental health continuum scores. Even if we are not in position to exclude mental components being emphasized by their medical and technical staff, diminishing related scores support the need for specialized mental health programs in elite sports and under circumstances of crises.

Interventions focused on stress or well-being within organizations have traditionally been couched within primary, secondary, and tertiary categories (see Fletcher et al., 2006). Specifically, these categories refer to interventions that (1) aim to reduce or prevent stressors from occurring (primary), (2) help individuals manage situations as they happen (secondary), or (3) promote reflection and learning about situations after they have happened (tertiary).

The above three points make perfect sense in relation to the attempted intervention. Infographic and the chosen online focus group sessions supported the initial goals of the program, with its results showing effectiveness on the psychological level ameliorating current contextual adversities. Intervention was also successful on preventing deterioration of quality of life and mental health indices in the participating athletes, a clear point of successfully completing the program based on the second category above. Finally, our intervention even if it showed efficacy, had to be short and concise due to its urgency and special conditions under which it had to run. Its short duration did not allow chances for detailed reflection on learning and relevant situations as they unraveled. It is always interesting to investigate the long-term effects of similar mental health interventions in the near future.

Both of our studies add to the scarce literature related to mental health in elite sports. They are in line with the proposals of the ISSP and FEPSAC consensus/position statements for improving mental health in sports (i.e., Henriksen et al., 2020) since they help not only with the assessment, but also with the implementation of a simple and clear plan to support mental health in an unprecedented human crisis.

Limitations of the program include the small number of participants in each group and the inability to manage adequately the comparison group. Both of those factors would not be there if the intervention had run under normal conditions. The seriousness and the immediacy of the situation and the perceived demand led us to design an intervention with existing resources, testing our reflexes, and readiness. Future attempts of a similar mental health sport psychology intervention program could take advantage of our study and expand it adequately. Cyclists participating in the comparison group followed mental health support recommendations by their technical and medical staff. Even if all cyclists competing at a national and international levels were invited to take part in the described intervention, only 38 cyclists decided to partake. Reported gender and competitive experience of these cyclists were not ideal. Future research attempts exploring the effects of similar interventions need to provide more clues on those factors enabling emphatic conclusions. Moreover, the use of a qualitative method of analysis could have contributed additional explanatory answers on this study. Another limitation of Study 2 relates to the nature of the study, which was quasi-experimental in nature, based on an intervention commissioned by ACCPI: it was based on voluntary participation and without randomization of its participants. Finally, generalization of the intervention results in elite sporting contexts demands a larger sample from various competitive sports.

We believe that this intervention creates an important first attempt to enhance mental health literacy in elite athletes facing a global pandemic and limited resources to retain their performance level. Through its description, analysis, and results, we provide an initial short framework of its kind. Overall, our attempt supports the foundation of evidence-based guidance for enhancing mental health awareness and implementing programs that acknowledge diversity and quality assurance in sport.

## Conclusion

Study 1 showed that the Mental Health Continuum questionnaire for sport has excellent validity and reliability also in an Italian sample of athletes. It can be a useful instrument for assessing the well-being of athletes. As highlighted by Uphill et al. (2016), it also provides “a conceptual space that encapsulates the broad spectrum of both distressing and flourishing experiences, but recognizes that the strategies designed to ameliorate distressing symptoms may not necessarily be the same as those designed to enhance flourishing” (p. 2). The fit indices of the Italian version are similar to the original version developed by Foster and Chow (2018) on the basis of the MHC-SF developed by Keyes (2002). However, the Italian sport sample shows differences in comparison to the sample of the general Italian population investigated by Petrillo et al. (2015).

Study 2 showed that our intervention (SPI) framed within an organizational setting can influence the psychological aspects of eudaimonic well-being for positive mental health (Keyes, 2007; Neil et al., 2017). Indeed, “the eudaimonic approach to well-being is not simply interested in subjective happiness, but in the realization of human potential” (Neil et al., 2017, p. 102). Our intervention focused on stress and well-being within organizations on secondary and tertiary categories (see Fletcher et al., 2006; Neil et al., 2017). Specifically, it helped individuals manage situations as they happened (secondary) and promoted reflection and learning from those situations after they occurred (tertiary).

## Data Availability Statement

The raw data supporting the conclusions of this article will be made available by the authors, without undue reservation.

## Ethics Statement

The studies involving human participants were reviewed and approved by IRB Bind Center University of Chieti-Pescara. The patients/participants provided their written informed consent to participate in this study.

## Author Contributions

All authors listed have made a substantial, direct and intellectual contribution to the work, and approved it for publication.

## Conflict of Interest

The authors declare that the research was conducted in the absence of any commercial or financial relationships that could be construed as a potential conflict of interest.

## Appendix 1

**Table:**

Sport MHC-SF During the past month, how often did your sport participant make you feel… *Nell'ultimo mese, quanto spesso la tua partecipazione allo sport ti ha fatto sentire…*	MHC-SF During the past month, how often did you feel… *Nell'ultimo mese quanto spesso ti sei sentito/hai sentito…*
1. Happy *Felice*	Happy *Felice*
2. Interested in your sport *Interessato al tuo sport*	Interested in life Interessato alla vita
3. Satisfied *Soddisfatto*	Satisfied with life *Soddisfatto*
4. That you had something to contribute to your team or sport community *Di poter dare un contributo alla tua squadra o alla tua comunità sportiva*	That you had something important to contribute to society *Di poter fare qualcosa di importante per la società*
5. That you belonged to your team or sport community *Di far parte della tua squadra o comunità sportiva*	That you belonged to a community (like a social group, or your neighborhood) *Parte di una comunità (un gruppo o il tuo quartiere)*
6. That your team or sport community is a good place for all participants *Che la tua squadra o comunità sportiva sono un buon ambiente per tutti coloro che ne fanno parte*	That your society is a good place, or is becoming a better place, for all people *Che la nostra società sta diventando u posto migliore per gente come te*
7. That people in your sport are basically good *Di avere a che fare con brave persone nel tuo sport*	That people are basically good *Che le persone sono fondamentalmente buone*
8. That the way your sport is organized makes sense to you *Che il modo in cui è organizzato il tuo sport ha senso per te*	That the way our society works makes sense to you *Che il modo in cui funziona la nostra società ha un senso per te*
9. That you liked most parts of your athletic personality *Di apprezzare la maggior parte degli aspetti della tua identità sportiva*	That you liked most parts of your personality *Che ti piacciono la maggior parte degli aspetti della tua personalità*
10. Good at managing the daily responsibilities of your sport *Di riuscire a gestire bene le responsabilità quotidiane del tuo sport*	Good at managing the responsibilities of your daily life *Bene nel gestire le responsabilità della tua vita quotidiana*
11. That you had warm and trusting relationships with others in your sport *Di avere, all'interno del tuo sport, relazioni cordiali e di fiducia con gli altri*	That you had warm and trusting relationships with others *Di avere delle relazioni sincere e cordiali con gli altri*
12. That you had sport experiences that challenged you to grow and become a better person *Di avere avuto esperienze sportive stimolanti per crescere e diventare una persona migliore*	That you had experiences that challenged you to grow and become a better person *Di aver avuto delle esperienze che ti hanno aiutato a crescere e a diventare una persona migliore*
13. Confident to think or express your own ideas and opinions to people in your sport *Fiducioso nel pensare o esprimere le tue idee e opinioni alle persone nel tuo sport*	Confident to think or express your own ideas and opinions *Capace di pensare o esprimere le tue idee e opinioni*
14. That you have a sense of direction or meaning within your sport *Che il tuo sport ha un senso o un significato per te*	That your life has a sense of direction or meaning to it *Che la tua vita ha un senso*
